# Predictive Method for Correct Identification of Archaeological Charred Grape Seeds: Support for Advances in Knowledge of Grape Domestication Process

**DOI:** 10.1371/journal.pone.0149814

**Published:** 2016-02-22

**Authors:** Mariano Ucchesu, Martino Orrù, Oscar Grillo, Gianfranco Venora, Giacomo Paglietti, Andrea Ardu, Gianluigi Bacchetta

**Affiliations:** 1 Centro Conservazione Biodiversità (CCB), Dipartimento di Scienze della Vita e dell’Ambiente (DISVA), Università degli Studi di Cagliari, V.le S. Ignazio da Laconi 11–13, 09123, Cagliari, Italy; 2 Dipartimento di Storia, Beni Culturali e Territorio–Sezione Archeologia, Università degli Studi di Cagliari, Piazza Arsenale 1–09124, Cagliari, Italy; 3 Stazione Consorziale Sperimentale di Granicoltura per la Sicilia, Via Sirio 1, 95041, Borgo San Pietro–Caltagirone (CT), Italy; 4 Dipartimento di Scienze Chimiche e Geologiche, Università degli Studi di Cagliari, Cittadella Universitaria, S.P. Monserrato-Sestu km 0.7, Monserrato, Italy; ISA, PORTUGAL

## Abstract

The identification of archaeological charred grape seeds is a difficult task due to the alteration of the morphological seeds shape. In archaeobotanical studies, for the correct discrimination between *Vitis vinifera* subsp. *sylvestris* and *Vitis vinifera* subsp. *vinifera* grape seeds it is very important to understand the history and origin of the domesticated grapevine. In this work, different carbonisation experiments were carried out using a hearth to reproduce the same burning conditions that occurred in archaeological contexts. In addition, several carbonisation trials on modern wild and cultivated grape seeds were performed using a muffle furnace. For comparison with archaeological materials, modern grape seed samples were obtained using seven different temperatures of carbonisation ranging between 180 and 340ºC for 120 min. Analysing the grape seed size and shape by computer vision techniques, and applying the stepwise linear discriminant analysis (LDA) method, discrimination of the wild from the cultivated charred grape seeds was possible. An overall correct classification of 93.3% was achieved. Applying the same statistical procedure to compare modern charred with archaeological grape seeds, found in Sardinia and dating back to the Early Bronze Age (2017–1751 2σ cal. BC), allowed 75.0% of the cases to be identified as wild grape. The proposed method proved to be a useful and effective procedure in identifying, with high accuracy, the charred grape seeds found in archaeological sites. Moreover, it may be considered valid support for advances in the knowledge and comprehension of viticulture adoption and the grape domestication process. The same methodology may also be successful when applied to other plant remains, and provide important information about the history of domesticated plants.

## Introduction

Plant remains can give important information about crop evolution, the origins of agriculture, the process of domestication, ancient plant economies and knowledge of past diet [1, 2, 3]. Commonly, these remains were preserved in archaeological sites in a carbonisation state; there are also special cases of different states of conservation such as waterlogged, dried or frozen [4]. Carbonisation represents one of the processes by which these remains suffer physical and chemical decomposition so preserving them from microbial attack [5]. In particular, the carbonisation process can be described as the exposure of plant materials to any heat source in the absence of air, determining a change to charcoal of the plant organic compounds [6, 7]. Such conditions are often observed in prehistoric hearths where it is more likely to find plant remains connected with cooking activities or the burning of waste products of plant origin [3–5, 8–10].

Plants remains may include, charcoal, fruits and seeds. In archaeobotanical studies, archaeological seeds can be identified at genus level when observable diagnostic characters remain [4]. In some cases, identification of these remains is not easy because of the alteration of the morphological seed shape [5, 11–13]. In fact, during the carbonisation process, different variables, such as temperature, exposure time, anoxic condition, chemical composition and seed water content, can modify the original morphology of the seeds [6, 14–19].

Moreover, since the archaeological seeds of many domesticated plants are morphologically very similar to those of wild ancestors, it is very difficult to distinguish them [5, 12]. For example, the seeds of *Vitis vinifera* subsp. *sylvestris* (C.C. Gmel.) Hegi and *Vitis vinifera* L. subsp. *vinifera* (hereafter called wild and cultivated grape), are highly similar. This phenotypic affinity would seem much more marked in carbonised seeds [20–22].

The grape is one of the most ancient and precious fruit-bearing plants in the Old World, and together with olive, fig, pomegranate and date palm, constitutes the oldest cultivated fruit trees [23]. The many grape seeds found in archaeological sites in the Near East region set the beginning of viticulture between the 3^rd^ and the 4^th^ millennium BC [3, 24]. Since the Early Bronze Age (EBA), the grape has played a role of primary importance in the complex societies of the Mediterranean Basin [3, 23, 25–27]. Many times, the origin of viticulture has been connected to the emergence of Bronze Age societies, due to their major territorial control and agricultural capabilities [3, 24, 28]. However, to date, the Mediterranean area has been subjected to scientific debate about the exact time and place of the origin of viticulture [3]. To this end, the chance to identify charred grape seeds found in archaeological sites could be particularly useful in understanding the time and place of the adoption of the cultivated grape [20]. However, the comparative complexity of discrimination between archaeological wild or cultivated grape seeds is linked to the preservation state of the remains, frequently found as charred [21].

In the past, Stummer’s index [29] was used to discriminate between wild and cultivated grape seeds found in archaeological sites [30–33]. In any event, several authors, investigating the carbonisation effects on grape seed morphology, have suggested that Stummer’s index appears unreliable when applied to charred seeds, because their original morphology has been irretrievably altered [5, 20, 21, 34, 35]. Therefore, carbonisation experiments conducted by Logothetis [34, 35] have highlighted the morphological changes that occurred in the wild and cultivated grape seeds during this chemo-physical process. In particular, he underlined the reduction in size to be related to the seed length and width but not to the thickness, and this effect was strongly marked in wild grape.

Likewise, Smith and Jones [21] conducted carbonisation experiments of cultivated grape seeds applying different temperatures, times of exposure to heat, and amounts of moisture and oxygen, underlining that Stummer’s index is not relevant for charred grape seeds, because of the remarkable increase in the breadth:length (B:L) index when subjected to charring at high temperatures [21].

Mangafa and Kotsakis conducted carbonisation trials on wild and cultivated grape seeds, measuring 12 morphometric features and verifying which of these were the most appropriate for the discrimination of the two subspecies [36]. Their work led to the conclusion that the most predictive features were length (*L*), length of the stalk (*LS*) and distance from the base of the chalaza to the tip of the stalk (*PCH*). Therefore, they proposed five mathematical indices, based on the three morphometric features (*L*, *LS*, *PCH*, *LS/L*, *PCH/L*), to identify charred archaeological seeds with an admissible error margin. These indices have been successfully applied to the archaeological grape seeds found in Dikili Tash and Toumba Thessaloniki in Greece, dating back to between the Late Neolithic and EBA [36, 37].

In recent years, the use of linear discriminant analysis (LDA) has allowed the discrimination of wild and cultivated seed species. Furthermore, LDA has discriminated seeds of different cultivar among one species [38–45]. Currently, this is considered a valid and non-destructive method, also commonly used to define the grape subspecific identity and verify the state of grape domestication in archaeobotanical studies [22, 44, 46–48].

Terral et al. [46] investigated the state of grape domestication in relation to the archaeological seeds found in Southern France, dating back to the first and second centuries. This study was carried out by geometrical analysis of the grape seed structure through Elliptic Fourier Descriptors (EFDs). The authors were able to establish the existence of local domestication processes during that period in the Languedoc region.

Likewise, Orrú et al. [44] and Ucchesu et al. [47] studied the state of grape domestication, analysing the seeds found in the Nuragic site of Sa Osa (Central West Sardinia), dating back to between the Middle and Late Bronze Age (LBA) (1391–1088 2σ cal. BC). Applying image analysis techniques and the LDA method, the authors concluded that during the LBA, primitive viticulture was already present in Sardinia.

Using the same discriminant analysis, Bouby et al. [22] showed that, in the South of France, the domestication processes of the grapevine was still in progress during the Roman period.

In all these works, these comparative methods were satisfactorily applied exclusively to uncharred waterlogged grape seeds. They are excellent samples to compare with modern materials because they do not exhibit the typical morphological deformations of the charred seeds [22, 44, 47], whereas the current method has not yet been applied to charred grape seeds. For this reason, three carbonisation experiments were conducted with the aim of studying the morphological variation of charred wild and cultivated grape seed and, using the LDA method, attempting the identification of archaeological charred seeds.

Therefore, the aims of this study are:

- to investigate the temperature range of carbonisation of modern grape seeds, using a hearth under anoxic conditions;
- to perform carbonisation experiments, on modern wild and cultivated grape seeds, at different temperatures and time exposures, using a muffle furnace;
- to verify the possibility of distinguishing charred wild and cultivated grape seeds, applying the LDA method;
- to acquire and analyse grape seed digital images to build a new database suitable for the identification of archaeological charred grape seeds;
- to validate the method of discriminating archaeological charred grape seeds dating back to the EBA.

## Materials and Methods

### Seed lot details

#### Modern samples

**Fig 1:**
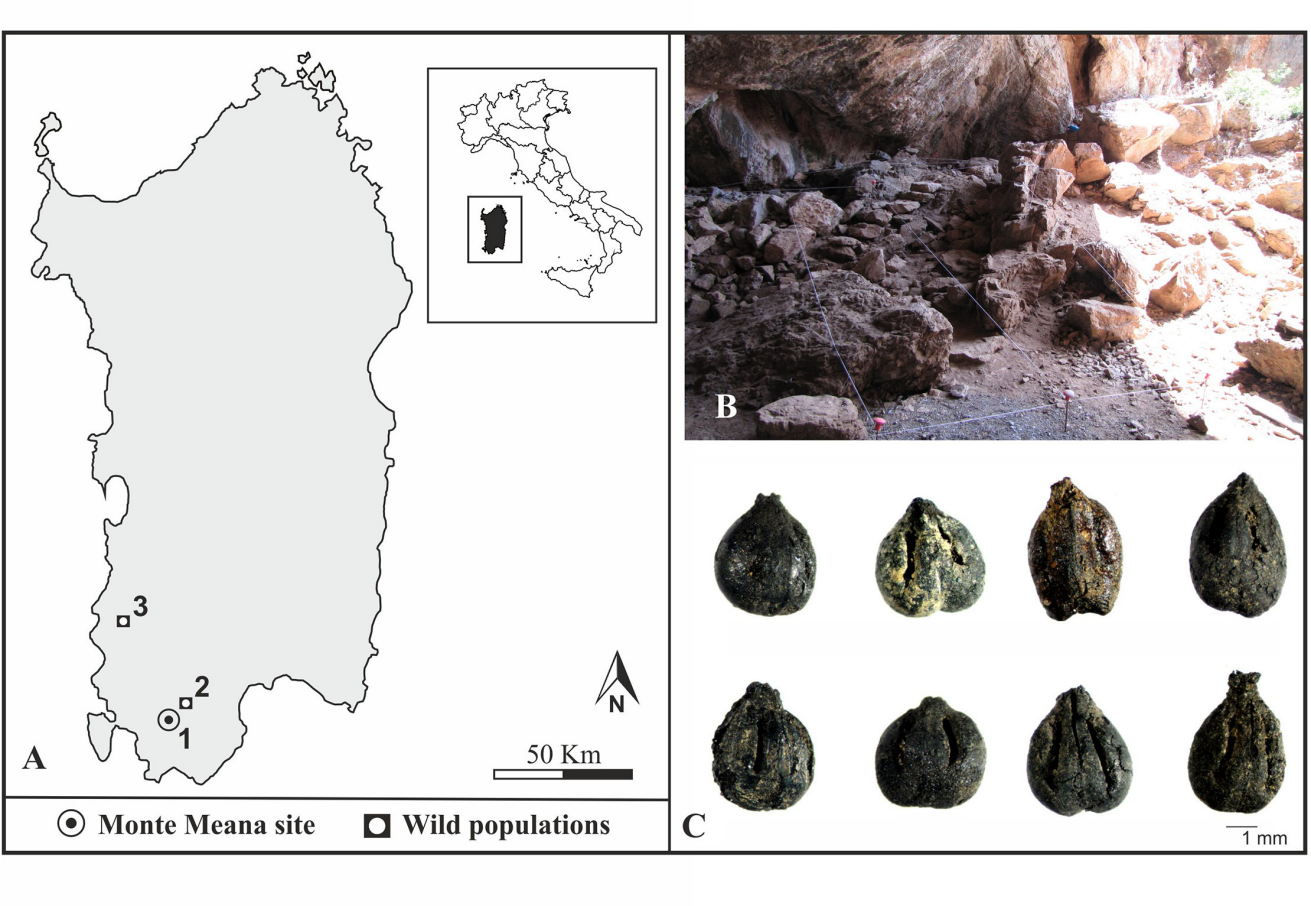
**A:** Location of the Monte Meana cave (1) and populations of *V*. *vinifera* subsp. *sylvestris* from Santadi (2) and Fluminimaggiore (3); **B:** Archaeological area of Monte Meana; **C:** Archaeological charred grape seeds discovered.

The wild grape seed samples used for the carbonisation experiments were collected along riverbanks and hilly humid slopes from two natural populations of Southern Sardinia (Fig 1).

The wild status of grapevine is confirmed by the study of flowers. The female flower is characterized by reflex stamens and infertile pollen that does not germinate, while the male flower has an underdeveloped modified carpel.

For the collected wild grape seed samples no specific permissions were required for these locations and activities and the field studies did not involve endangered or protected species.

The wild grape seeds were stored according to the manual for the *ex situ* conservation of wild plants [49].

Cultivated grape, representing the traditional varieties currently cropped in Sardinia, were selected from the AGRIS germplasm collections (Agenzia per la Ricerca in Agricoltura della Regione Sardegna) of Ussana (Sardinia, Italy) (Table 1).

**Table 1:** Accession code, grape variety, taxon (Vs: *V*. *vinifera* subsp. *sylvestris*; Vv: *V*. *vinifera* subsp. *vinifera*), grape colour (B: black; W: white) and charred method. In parenthesis the numbers of fresh seeds utilised in the different carbonisation experiments.

Code	Variety name	*Taxon*	Grape colour	Hearth one	Muffle furnace	Hearth two		Number of seeds
						1°	2°	
FLU	Fluminimaggiore	Vs	B	x (150)	x (210)	x (30)	x (1,000)	1,390
SAN	Santadi	Vs	B	x (150)	x (210)	x (30)	x (1,000)	1,390
CAN	Cannonau nero di Sestu	Vv	B	x (150)	x (210)	x (30)		390
GRA	Granazza di Garaumele	Vv	W	x (150)	x (210)	x (30)		390
CAR	Carenisca	Vv	B		x (210)			210
VER	Vertudi	Vv	B		x (210)			210
NUR	Nuragus	Vv	W		x (210)			210
REM	Remungiau di Serri	Vv	W		x (210)			210
AXI	Axina de tres bias	Vv	B				x (142)	142
MOS	Moscato nero di Ulatirso	Vv	B				x (142)	142
NAS	Nasco nero di Abbasanta	Vv	B				x (142)	142
GIO	Gioia bella di Serramanna	Vv	B				x (142)	142
CRG	Carignano	Vv	B				x (142)	142
MAN	Manzesu	Vv	B				x (142)	142
PRI	Primidivu nieddu di Padria	Vv	B				x (142)	142
NER	Nera di Bosa	Vv	B				x (142)	142
SAL	Saluda e passa	Vv	B				x (142)	142
AMI	Amiga di Lanusei	Vv	B				x (142)	142
NIE	Nieddu mannu di Nurri	Vv	B				x (142)	142
MON	Monica di Murri	Vv	B				x (142)	142
MUR	Muristellu bovale Triei	Vv	B				x (142)	142
MSE	Moscatello nero di Seulo	Vv	B				x (142)	142
CBO	Cannonau bianco Oliena	Vv	W				x (142)	142
BIA	Bianca di Serri	Vv	W				x (142)	142
MPA	Moscato di Patada	Vv	W				x (142)	142
BIP	Bianca di Padria	Vv	W				x (142)	142
APE	Apesorgia bianca	Vv	W				x (142)	142
LIC	Licronaxiu Nuraxinieddu	Vv	W				x (142)	142
GRI	Grillu	Vv	W				x (142)	142
LIC	Licronaxiu	Vv	W				x (142)	142
COD	Codronisca	Vv	W				x (142)	142
ALI	Alicante	Vv	W				x (142)	142
ARG	Argumannu	Vv	W				x (142)	142
RET	Retagliadu di Monti	Vv	W				x (142)	142
SEM	Semidano Murri	Vv	W				x (142)	142
VER	Vernaccia di Escalaplano	Vv	W				x (142)	142
Total								8,376

#### Archaeological samples

The archaeological samples were found in the cave of Monte Meana (Santadi) (39°2'28"N 8°42'31"E, 166 m a.s.l.), in the Sulcis region (South-Western Sardinia) (Fig 1A). This site belongs to a region of high density of archaeological sites, where human occupation has been testified since the Middle Neolithic (5^th^ millennium BC) [50–52]. The karst cave opens on the south-western side of the limestone massif of Monte Meana, where several structures, such as stone walls, stairs and hearths, have been identified inside (Fig 1B) [50]. In one of these hearths, on a layer of ashes, charcoals and burnt animal bones, a large amount of fragments of cooking vessels and eight charred grape seeds were discovered. The latter are the object of this study (Fig 1C). Radiocarbon dating, of the charcoals collected from the hearth (LTL4198A: 2017–1751 2σ cal. BC) revealed the use of this area during the EBA [50–52]. No permits were required for the described study, which complied with all relevant regulations.

### Heat treatment

For this study, three different carbonisation experiments were conducted. The main goal was to obtain seed samples as similar as possible to the archaeological charred grape seeds. So, according to the experiments conducted by Smith and Jones [21] and Mangafa and Kotsakis [36] all the carbonisation experiments has been performed under anoxic conditions. The anoxic conditions was ensured by covering the seeds with topsoil. Therefore, the first carbonisation experiment was allowed to investigate temperature and exposure time under anoxic conditions of the modern grapes seeds both wild and cultivated, through an experimental hearth. The second experiment was conducted using a muffle furnace in which temperature and exposure time were established following the results obtained with the first experiment. The final experiment was conducted carbonising the grape seeds through a simulation of the common prehistoric conditions of carbonisation, using a hearth on the ground. The charred modern samples obtained with the different experiments were compared with the archaeological charred grape seeds. Hereafter, detailed procedures are given to explain the experimental methodology of the three carbonisation experiments.

#### Measurements of thermal carbonisation

Thermal carbonisation was performed using an experimental hearth (hereafter hearth 1), with the aim of investigating the thermal carbonisation of wild and cultivated grape seeds under anoxic conditions.

**Fig 2:**
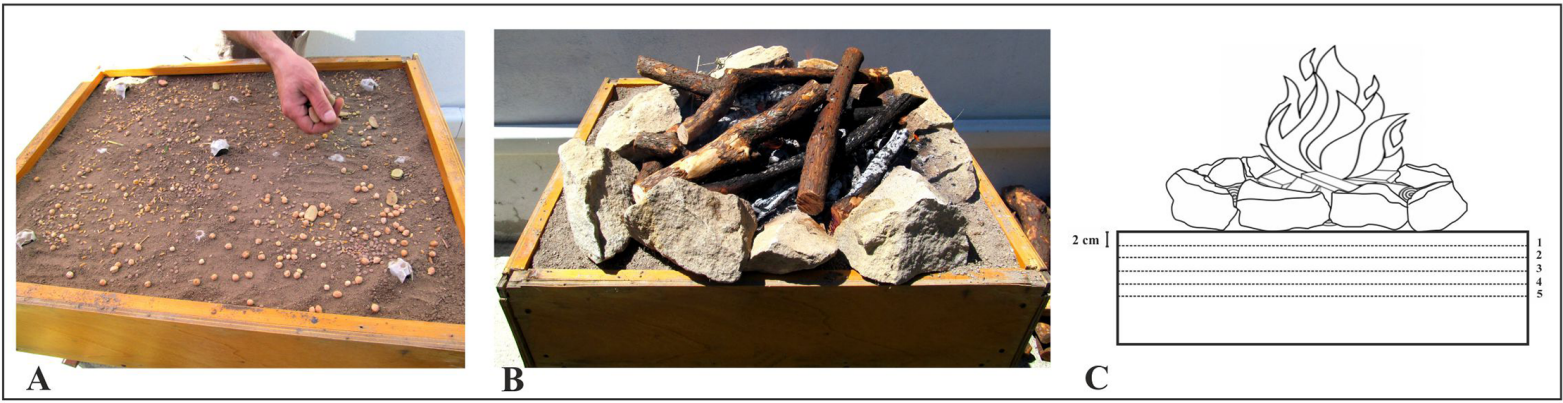
**A**: Reconstruction of hearth 1; **B**: lighted; **C**: section reconstruction of the five layers in which were deposited the seeds.

Hearth 1 was built using a box, 720×640×200 mm, filled with topsoil and overlapping a circle of stones, reducing the available area to 500×500 mm (Fig 2A and 2B). The first 100 mm of topsoil were divided into five layers of 20 mm each, where 30 seeds, of the wild (FLU, SAN) and cultivated grape (CAN, GRA) (Table 1), were placed in each layer. Each layer was labelled with an identification number, increasing from the top to the bottom (Fig 2C). For the experiment, 100 Kg of natural wood characteristic of the area (*Quercus ilex* L., *Arbutus unedo* L. and *Erica arborea* L.) were burned. The thermal measurements were taken on each layer using a thermocouple (Testo model 925) with a probe of 20 cm, introduced from the bottom of the box, through holes that directly led to the five layers.

Temperature measurements were recorded every 30 min; therefore, the whole experiment was 600 min long. After 24 h, when the soil was completely cooled, the layers were removed with a trowel and the seeds were collected.

#### Heat treatment under constant oven temperatures

Carbonisation experiments under constant oven temperatures were performed using a muffle furnace (Lenton furnaces model ARF 7/22). The grape seeds used in this experiment consisted of two wild (FLU, SAN) (Table 1) and six cultivated grape variety samples, three black (CAN, CAR, VER) and three white (NUR, REM, GRA) (Table 1). For each lot, 30 seeds were treated.

The anoxic conditions were ensured by placing grape seeds in aluminium trays with a 2-cm cover of topsoil. According to the temperature generated in the hearth 1 carbonisation experiment, seven temperatures were specifically tested on each seed lot: 180, 200, 220, 240, 290, 310 and 340°C. The seeds were heated for 120 min for each temperature. According to Braadbaart [16], a ramp rate of 2°C min^-1^ was set until reaching the final temperature.

#### Heat treatment under uncontrolled thermal conditions

**Fig 3:**
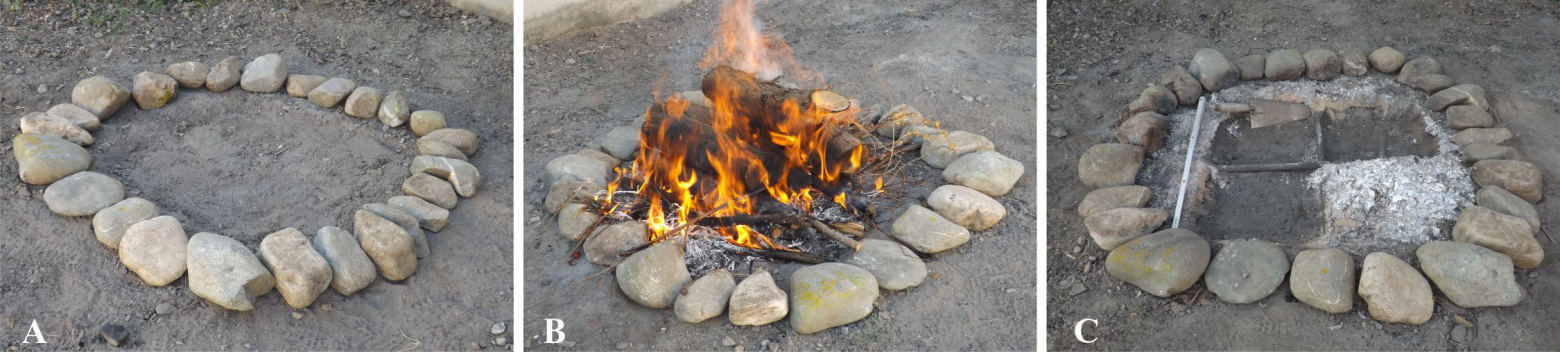
Representation of the operating sequence: **A**: reconstruction of hearth 2; **B**: lighted; **C**: samples collection.

A carbonisation experiment under uncontrolled thermal conditions was also carried out using a hearth on the ground (hereafter hearth 2), simulating the normal prehistoric conditions of carbonisation (Fig 3). The hearth was built on a pit in the ground 30 cm deep (Fig 3A). It was 1 m in diameter and surrounded by stones, divided into four equal-sized areas (Fig 3A). Also in this case, the grape seeds were covered with 2 cm of topsoil, reproducing the same anoxic conditions established for hearth 1. For this experiment 200 Kg of natural wood were burned (Fig 3B).

The carbonisation experiment involved four seed lots: two cultivated (CAN, GRA), and two wild (FLU, SAN) (Table 1). For each lot, 30 seeds were treated. To facilitate the collection of seeds after the trial, each grape variety was placed in one of four areas of the hearth (Fig 3C). The hearth was kept burning for 10 h and continuously fuelled with new wood. At the end of this burning cycle, the hearth was extinguished and, after 24 h, when the ground was completely cooled, the grape seeds were collected (Fig 3C). In the second step, following the same procedure, we proceeded to carbonise 2,000 wild and 2,000 black and 2,000 white cultivated grape seeds respectively (Table 1).

### Data analysis

#### Digital image analysis

**Fig 4:**
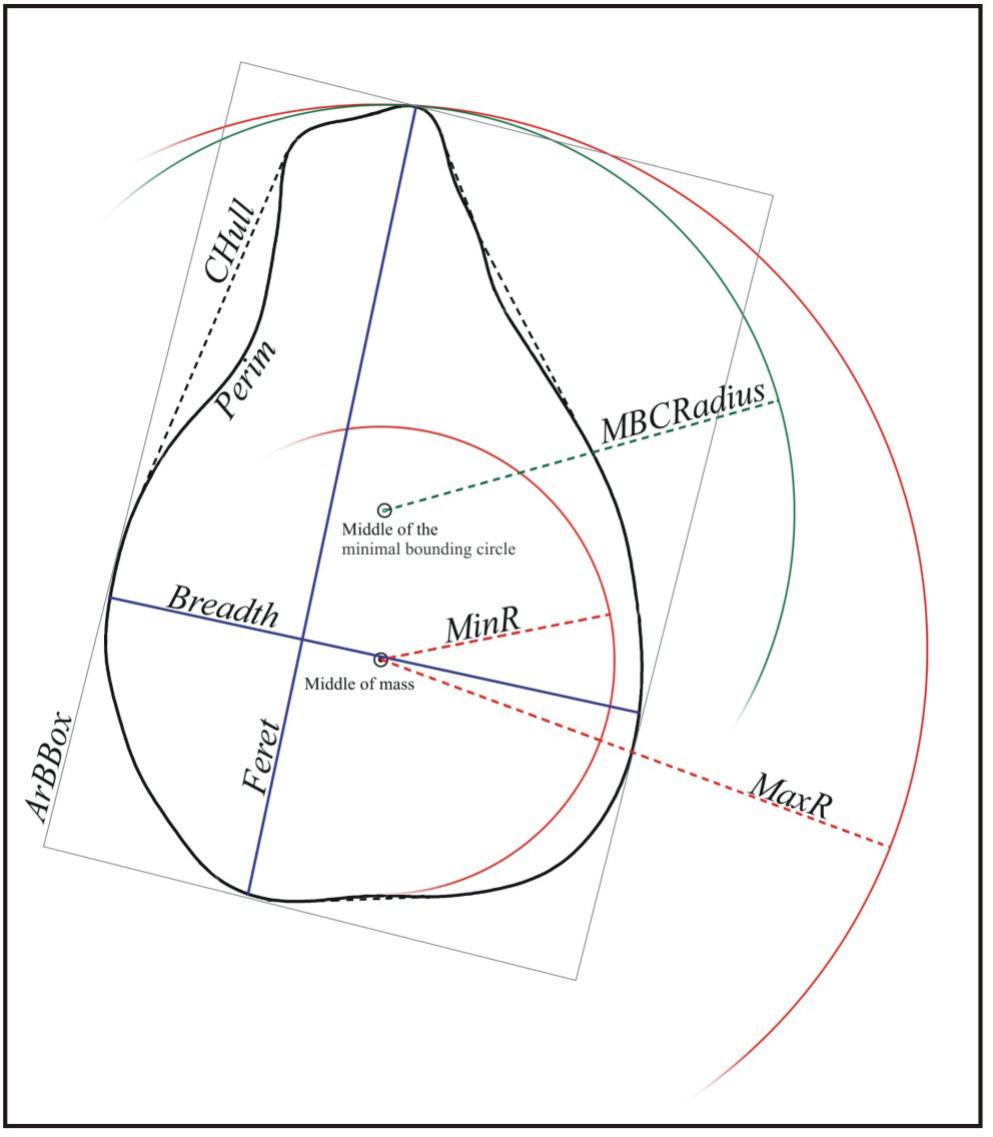
Graphic representation of some morphometric measurements.

Following the same methodology used by Orrù et al. [44, 45] and Ucchesu et al. [47], digital images of charred and untreated grape seeds were acquired using a flatbed scanner (Epson Perfection V550), with a digital resolution of 400 dpi for a scanning area not exceeding 1024×1024 pixels. In order to represent the whole variability of the grape seed lots, the samples were scanned twice exposing them on the ventral and dorsal sides. The images were processed and analysed using ImageJ v. 1.49 (http://rsb.info.nih.gov/ij). A plugin, *Particles8* [53], freely available on the official website (http://www.mecourse.com/landinig/software/software.html) was used to measure 26 seed morphometric features (Table 2, Fig 4). A further 80 Elliptic Fourier Descriptors (EFDs) [44–47], descriptive of the seed contour shape, were computed using the open source SHAPE software [54], increasing the number of discriminant parameters [44, 45, 54–56]. This method allows description of the boundary of the seed projection as an array of complex numbers which correspond to the pixel positions on the seed boundary. So, from the seed apex, defined as the starting point in a Cartesian system, chain codes are generated. A chain code is a lossless compression algorithm for binary images. The basic principle of chain codes is to separately encode each connected component (pixel) in the image. The encoder then moves along the boundary of the image and, at each step, transmits a symbol representing the direction of this movement.

**Table 2:** List of the 26 morphometric seed features measured.

Parameter	Description
*Perim*	Perimeter, calculated from the centres of the boundary pixels
*Area*	Area inside the polygon defined by the perimeter
*Pixels*	Number of pixels forming the seed image
*MinR*	Radius of the inscribed circle centred at the middle of mass
*MaxR*	Radius of the enclosing circle centred at the middle of mass
*Feret*	Largest axis length
*Breadth*	Largest axis perpendicular to the Feret
*CHull*	Convex hull or convex polygon calculated from pixel centres
*CArea*	Area of the convex hull polygon
*MBCRadius*	Radius of the minimal bounding circle
*AspRatio*	Aspect ratio = Feret/Breadth
*Circ*	Circularity = 4·π·Area/Perimeter^2^
*Roundness*	Roundness = 4·Area/(π·Feret^2^)
*ArEquivD*	Area equivalent diameter = √((4/π)·Area)
*PerEquivD*	Perimeter equivalent diameter = Area/π
*EquivEllAr*	Equivalent ellipse area = (π·Feret·Breadth)/4
*Compactness*	Compactness = √((4/π)·Area)/Feret
*Solidity*	Solidity = Area/Convex_Area
*Concavity*	Concavity = Convex_Area-Area
*Convexity*	Convexity = Convex_hull/Perimeter
*Shape*	Shape = Perimeter^2^/Area
*RFactor*	RFactor = Convex_Hull /(Feret·π)
*ModRatio*	Modification ratio = (2·MinR)/Feret
*Sphericity*	Sphericity = MinR/MaxR
*ArBBox*	Area of the bounding box along the feret diameter = Feret·Breadth
*Rectang*	Rectangularity = Area/ArBBox

This continues until the encoder returns to the starting position. This method is based on separate Fourier decompositions of the incremental changes of the X and Y coordinates as a function of the cumulative length along the boundary [57]. Each harmonic (*n*) corresponds to four coefficients (a*n*, b*n*, c*n* and d*n*) defining the ellipse in the XY plane. The coefficients of the first harmonic, describing the best fitting ellipse of outlines, are used to standardize size (surface area) and to orientate seeds [46]. According to many authors [56, 58, 59, 60], about the use of a number of harmonics for an optimal description of seed outlines, 20 harmonics were used to define the seed boundaries, obtaining a further 77 parameters useful to discriminate among the studied seeds [44]. Overall, 103 morphometric variables were measured.

#### LDA

The measured parameters were used to build a database of features descriptive of seed size and shape. Using the SPSS software package release 16.0 (SPSS 16.0 for Windows; SPSS Inc., Chicago, IL), the data were statistically elaborated applying the stepwise LDA to compare the charred wild and cultivated grape seed lots and the archaeological charred grape seeds.

This is a well-known method, commonly applied to reduce the dataset dimensions without losing significant information and to classify the statistical cases into groups [61–65]. The optimal projection or transformation in classical LDA is obtained by finding the combination of predictor variables with the aim of minimising the within-class distance and maximising the between-class distances simultaneously, thus achieving maximum class discrimination [64, 66, 67]. The stepwise method identifies and selects the most statistically significant features, among the 103 measured on each seed, using three statistical variables: *Tolerance*, *F*-to-enter and *F*-to-remove. The *Tolerance* value indicates the proportion of variable variance not accounted for in other independent variables in the equation. *F*-to-enter and *F*-to-remove values define the power of each variable in the model and are useful in describing what happens if a variable is inserted and removed, respectively, from the current model. This selective process starts with a model that does not include any of the original morphometric variables. At each step, the variable with the largest *F*-to-enter value, that exceeds the entry criterion chosen (*F* ≥ 3.84), is added to the model. The variables left out of the analysis at the last step have *F*-to-enter values smaller than 3.84, and, therefore, no more are added. The process is automatically stopped when no remaining variables are able to increase the discrimination ability [39]. Finally, a cross-validation procedure is applied to verify the performance of the identification system, testing individual unknown cases and classifying them on the basis of all others. This procedure, also called rotation estimation [68, 69], was applied, both to evaluate the performance and to validate any implemented classifier. The validation procedure used here is the leave-one-out cross-validation (LOOCV). It involves using a single case from the original sample set as the validation dataset, and the remaining cases as the training set. Each case is classified into a group according to the classification functions computed from all the data except the case being classified. The proportion of misclassified cases after removing the effect of each case one at a time is the leave-one-out estimate of misclassification [70].

## Results

### Heat treatment

#### Measurements of thermal carbonisation by hearth 1

**Fig 5:**
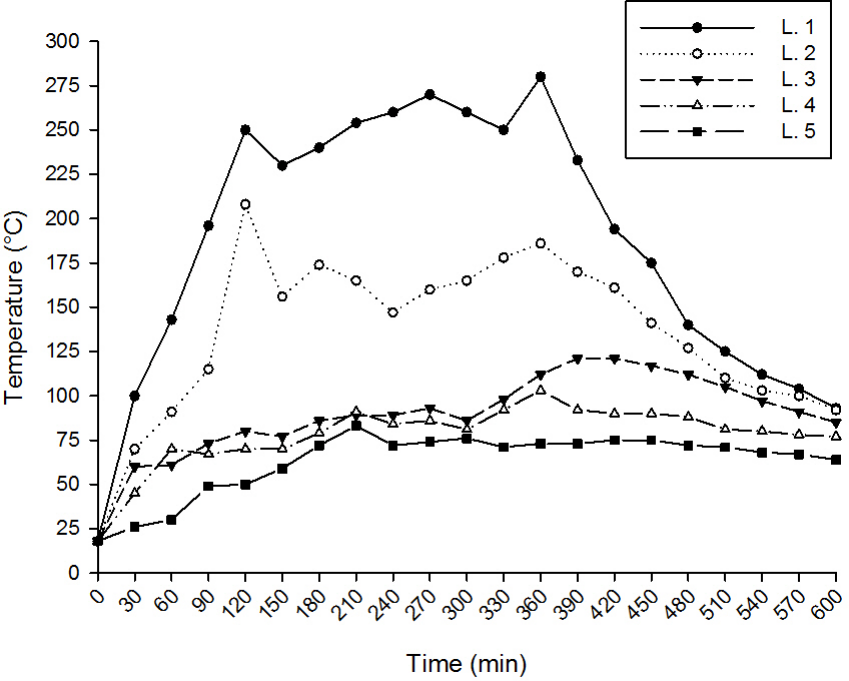
Temperatures recorded in the heat treatment (hearth 1) for the five layers of soil during the entire experiment.

Measurements of thermal carbonisation carried out in the five layers of hearth 1 showed that after 30 min from ignition layer one with an initial temperature of 27°C reached a temperature of 100°C. At 120 min the recorded temperature was 250°C and it was maintained for a further 240 min (Fig 5). At the end of the trial, the temperature decreased to below 100°C (Fig 5).

Following the same trend as layer one, but reaching temperatures considerably lower, layer two recorded a little more than 200°C after 120 min, decreasing to 160°C and staying quite steady for a further 240 min (Fig 5). Similar to layer one, at the end of the trial, the temperature decreased to below 100°C (Fig 5).

The highest temperature recorded in layer one was 280°C and in layer two 208°C, while in the others the highest temperatures reached were between 83 and 121°C, insufficient to carbonise the seeds (Fig 5).

**Fig 6:**
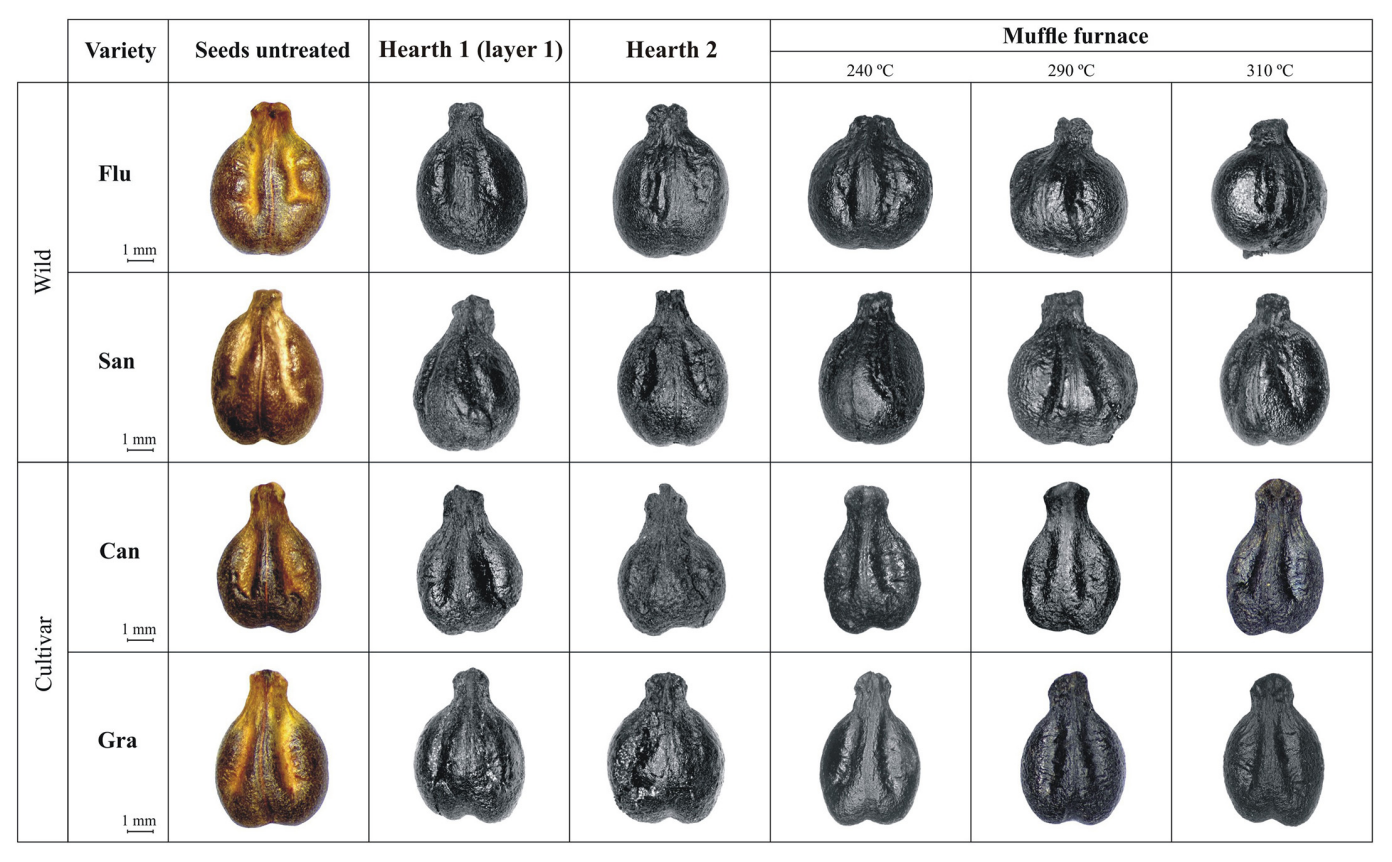
Samples of untreated and charred grape seeds carbonised using different temperatures and carbonisation experiments.

According to the achieved results, all the seeds put in layer one were totally charred; those in layer two were partially charred; while all the seeds arranged in the others layers did not suffer any process of carbonisation (Fig 6).

#### Charring seeds under constant oven temperatures by muffle furnace

The carbonisation experiment using the muffle furnace showed that the seeds subjected to a controlled temperature, in the range between 180°C and 220°C, were not charred, whereas, the seeds subjected to the highest temperatures, 240, 290 and 310°C, showed homogeneous carbonisation and no protrusions or deformations were generated (Fig 6). Finally, the seeds treated at temperatures of 340°C were wholly incinerated.

According to the results obtained in this charring trial, morphological analyses were conducted exclusively on the seeds totally carbonised at temperatures of 240, 290 and 310°C.

#### Charring seeds by hearth 2

The carbonisation experiment conducted with hearth 2 allowed a high amount of charred grape seeds to be obtained quickly. Thanks to the previous experiment conducted with hearth 1, it was possible to establish a minimum depth of 2 cm in which to bury the seeds in the ground. So, from hearth 2 a total of 1,890 intact charred wild and 3,800 cultivated grape seeds were recovered. Also in this case, morphological analyses were conducted using exclusively the seeds totally carbonised and intact (Fig 6).

#### Discriminant analysis

In order to prove the actual morphological differentiation between wild and cultivated grape seeds, a preliminary comparison among the modern untreated wild and cultivated grape seed lots was executed, achieving an overall percentage of correct identification of 90.4% (Table 3).

**Table 3:** Correct classification percentage between untreated *V*. *vinifera* subsp. *vinifera* and *V*. *vinifera* subsp. *sylvestris* and charred (in muffle furnace) *V*. *vinifera* subsp. *vinifera* and *V*. *vinifera* subsp. *sylvestris* seed lots. In parenthesis the numbers of analysed seeds (u) = untreated; (c) = charred.

	*V. vinifera* subsp. *vinifera*	*V. vinifera* subsp. *sylvestris*	Total
*V*. *vinifera* subsp. *vinifera* (u)	**94.4 (775)**	5.6 (46)	100 (821)
*V*. *vinifera* subsp. *sylvestris* (u)	17.6 (71)	**82.4 (333)**	100 (404)
Overall		** **	**90.4% (1,225)**
*V*. *vinifera* subsp. *vinifera* (c)	**98.0 (1055)**	2.0 (21)	100 (1,076)
*V*. *vinifera* subsp. *sylvestris* (c)	25.0 (90)	**75.0 (270)**	100 (360)
Overall			**93.3% (1,436)**

A similar comparison was conducted among the two seed lots of charred wild (FLU, SAN) and cultivated grape seeds (CAN, CAR, GRA, NUR, REM, VER) using the muffle furnace. In this case, an overall correct classification of 93.3% was achieved (Table 4). To verify the separation between treated and untreated seeds, two further morphological comparisons were undertaken between the charred and untreated wild grape and charred and untreated cultivated grape seed lots, respectively (Table 4). The analysis showed high discrimination performance for both cases. Specifically, in the case of wild grape seeds the percentage of correct discrimination was of 96.9% (Table 4), whereas, in the case of the cultivated grape seeds the correct percentage of classification was 83.8% (Table 4).

**Table 4:** Correct classification percentage between charred (in muffle furnace) and untreated *V*. *vinifera* subsp. *sylvestris* and *V*. *vinifera* subsp. *vinifera* seed lots. In parenthesis the numbers of analysed seeds.

	Charred	Untreated	Total
	*V*. *vinifera* subsp. *sylvestris*		
Charred	**96.7 (348)**	3.3 (12)	100 (360)
Untreated	3 (12)	**97 (392)**	100 (404)
Overall			**96.9% (764)**
	*V*. *vinifera* subsp. *vinifera*		
Charred	**90 (968)**	10 (108)	100 (1,076)
Untreated	24.4 (200)	**75.6 (621)**	100 (821)
Overall			**83.8% (1,897)**

Afterwards, a discriminant analysis was implemented in order to compare the charred seeds of the wild and cultivated grapes carbonised at different temperatures. The discrimination analysis showed an overall correct classification of between 91.4% and 94.1%, confirming differentiation of these samples for all carbonisation temperatures (Table 5).

**Table 5:** Correct classification percentage between *V*. *vinifera* subsp. *vinifera* and *V*. *vinifera* subsp. *sylvestris* seed lots charred at different temperatures using the muffle furnace. In parenthesis the numbers of analysed seeds.

	*V*. *vinifera* subsp. *vinifera* 240 °C	*V*. *vinifera* subsp. *sylvestris* 240 °C	Total
*V*. *vinifera* subsp. *vinifera* 240 °C	**99.7 (357)**	0.3 (1)	100 (358)
*V*. *vinifera* subsp. *sylvestris* 240 °C	22.5 (27)	**77.5 (93)**	100 (120)
Overall			**94.1% (478)**
	*V*. *vinifera* subsp. *vinifera* 290 °C	*V*. *vinifera* subsp. *sylvestris* 290 °C	
*V*. *vinifera* subsp. *vinifera* 290 °C	**96.7 (348)**	3.3 (12)	100 (360)
*V*. *vinifera* subsp. *sylvestris* 290 °C	22.5 (27)	**77.5 (93)**	100 (120)
Overall			**91.9% (480)**
	*V*. *vinifera* subsp. *vinifera* 310 °C	*V*. *vinifera* subsp. *sylvestris* 310 °C	
*V*. *vinifera* subsp. *vinifera* 310 °C	**97.2 (348)**	2.8 (10)	100 (358)
*V*. *vinifera* subsp. *sylvestris* 310 °C	15.8 (19)	**84.2 (101)**	100 (120)
Overall			**93.9% (478)**

To verify the validity of the discriminant analysis, two seed lots of wild (FLU, SAN) and cultivated grapes (CAN, GRA), charred by hearth 2 and considered as unknown test datasets, were compared with seed lots carbonised using the muffle furnace. In this case, all seed lots considered as unknowns were correctly classified, reaching an overall correct classification of 92.3% (Table 6).

**Table 6:** Correct classification percentage among charred *V*. *vinifera* subsp. *vinifera* and *V*. *vinifera* subsp. *sylvestris* seed lots (in muffle furnace), and *V*. *vinifera* subsp. *vinifera* and *V*. *vinifera* subsp. s*ylvestris* seed lots, charred using hearth 2 and considered as unknown test datasets. In parenthesis the numbers of analysed seeds. (mf) = muffle furnace; (h2) = experimental hearth 2

	*V. vinifera* subsp. *vinifera*	*V. vinifera* subsp. *sylvestris*	Total
*V*. *vinifera* subsp. *vinifera* (mf)	98 (1,055)	2 (21)	100 (1,076)
*V*. *vinifera* subsp. *sylvestris* (mf)	25 (90)	75 (270)	100 (360)
Unknown cultivated grapevine (h2)	**81 (85)**	19 (20)	100 (105)
Unknown wild grape (h2)	18.6 (22)	**81.4 (96)**	100 (118)
Overall			**92.3% (1,659)**

Finally, in order to obtain significant information about the archaeological grape seeds from Monte Meana and understand whether or not they are more similar to the current wild or cultivated grapes, these archaeological seeds were compared with the wild and cultivated grape seed lots carbonised using the muffle furnace. In this case, the archaeological seeds, individually considered as an unknown group, showed similarities with the charred wild grapes in 81.3% of cases (Table 7). An additional comparison with the unknown archaeological seeds from Monte Meana was carried out in relation to the origin groups of the wild grapes collected from Fluminimaggiore and Santadi and charred in the muffle furnace. In this case, 68.8% of the cases was classified as charred wild grape from Santadi (Table 8). In addition, the largest wild (FLU, SAN) and 28 cultivated grape seed lots (Table 1) charred by hearth 2 in the second step, were tested. Also in this case, the unknown archaeological seeds had confirmed similarities with the charred wild grapes in 75.0% of cases (Table 9).

**Table 7:** Correct classification percentage between the archaeological seed lot from Monte Meana, considered as unknown group, and the two charred lots (in muffle furnace) of *V*. *vinifera* subsp. *vinifera* and *V*. *vinifera* subsp. *sylvestris*. In parenthesis the numbers of analysed seeds.

	*V. vinifera* subsp. *vinifera*	*V. vinifera* subsp. *sylvestris*	Total
*V*. *vinifera* subsp. *Vinifera*	98 (1,055)	2 (21)	100 (1,076)
*V*. *vinifera* subsp. *sylvestris*	25 (90)	75 (270)	100 (360)
Archaeological seeds Monte Meana	18.7 (3)	**81.3 (13)**	100 (16)
Overall			**92.3% (1,452)**

**Table 8:** Correct classification percentage between the archaeological seed lots from Monte Meana, considered as unknown group, and the two charred lots (in muffle furnace) of wild grape from Fluminimaggiore and Santadi. In parenthesis the numbers of analysed seeds.

	FLU	SAN	Total
FLU	81.1 (146)	18.9 (34)	100 (180)
SAN	18.3 (33)	81.7 (11)	100 (180)
Archaeological seeds Monte Meana	31.2 (5)	**68.8 (11)**	100 (16)
Overall			**81.4 (376)**

**Table 9:** Correct classification percentage between the archaeological seed lot from Monte Meana, considered as unknown group, and the two charred lots (in hearth 2) of *V*. *vinifera* subsp. *vinifera* and *V*. *vinifera* subsp. *sylvestris* (FLU and SAN). In parenthesis the numbers of analysed seeds.

	*V. vinifera* subsp. *vinifera*	*V. vinifera* subsp. *sylvestris*	Total
*V*. *vinifera* subsp. *vinifera*	98.5 (7,094)	1.5 (106)	100 (7,200)
*V*. *vinifera* subsp. *sylvestris*	12.3 (442)	87.7 (3,158)	100 (3,600)
Archaeological seeds Monte Meana	25.2 (4)	**75.0 (12)**	100 (16)
Overall			**94.9% (10,816)**

## Discussion

Carbonisation experiments carried out on the wild and cultivated grape seeds allowed investigation into the grape seed charring process, obtaining excellent samples for use as comparative materials for archaeological seed identification.

Comparative analysis between untreated and charred wild and cultivated grape seeds showed that the carbonisation process generates strong changes in the diagnostic features in both cases. So, the charred wild and cultivated grape seeds no longer appear similar to untreated grape seed lots. This achievement proves that it is not appropriate, in the archaeobotanical field, to compare charred seeds with untreated modern materials.

The results of the carbonisation experiment carried out with hearth 1, allowed investigation of the temperatures generated at different depths under the topsoil and verified the state of the carbonisation of the seeds. Furthermore, this experiment allowed the carbonisation methodology with the muffle furnace to be refined, with as much control as possible of the heat time exposure, temperature and anoxic conditions. In addition, this experiment has allowed the calibration of the best temperature and exposure time helpful in achieving optimal carbonisation of the grape seeds. The grape seeds charred by the muffle furnace showed that complete carbonisation occurred in a limited temperature range, between 240 and 310°C. The same results were achieved in layer one of hearth 1. Thus, the archaeological grapes seeds probably also follow the same carbonisation conditions that may be listed in this limited temperature range, for which lower or higher temperatures are not adequate for their preservation.

This may be useful in the archaeological field in understanding the taphonomic processes of plant remains. Furthermore, carbonisation experiments conducted with the muffle furnace have allowed charred seeds samples very similar to charred grape seeds to be obtained with the experimental hearths. This shows that we can obtain excellent comparison samples using experimental hearths. In this way, all the variables involved in the carbonisation process will be included if a large number of seeds is used.

Then, the efficiency of the methodological procedure was tested, comparing some accessions by hearth 2 of charred wild and cultivated grapes. The samples of charred wild and cultivated grape seeds, considered as unknown groups and compared with the grape seeds carbonised in the muffle furnace, were correctly classified by the discriminant analysis. The great amount of charred grape seeds obtained by hearth 2 allowed the built database to be reinforced, so improving the identification system and minimising misclassifications.

Finally, analysing the archaeological charred grape seeds from Monte Meana, adding them into the classification model as an unknown group, it was possible to obtain a plausibly correct identification of the seed lots as wild grape. In addition, the comparison between the archaeological seed lots from Monte Meana and the two seed lots of wild grapes collected from Fluminimaggiore and Santadi allowed identification, with good approximation, of this seed lot as modern wild grape from Santadi. Likewise, the archaeological grape seeds, added as unknown in the new charred grape seeds database, including the greater amount of wild and cultivated grape seeds, were confirmed to be wild grape.

According to these achievements, it is possible to assume that the grape seeds found in the archaeological site of Monte Meana belonged to the wild species collected near to the site and that currently still grow along river shores and on screeds (colluvial sites) of hilly humid slopes. This result shows that the wild grapes that still grow in the area of Santadi have not changed over time and that no outside influence has occurred in this territory. Probably, it is not a mere causality that Santadi is located in an area of Sardinia where the very low anthropisation did not affect the territory that remained unchanged for millennia [71].

The results obtained with the archaeological seeds of Monte Meana have also allowed the understanding that viticulture had not yet started in this area during the EBA. Instead, the new data obtained in recent work established that viticulture in Sardinia seemed to appear between the Middle and the LBA [49]. On the basis of this data, it is possible to hypothesise that probably, in Sardinia, the origins of viticulture should be sought between the EBA and LBA (ca. 2200–1150 BC).

## Conclusion

This work allowed the setting and testing of an efficient way to identify, with a low margin of error, archaeological charred grape seeds. Image analysis techniques, together with the most appropriate statistical approaches, can be considered a valuable tool in supporting the characterisation and recognition of archaeobotanical charred samples. Further carbonisation experiments, performed on a larger number of modern wild and cultivated grape seeds, have to be conducted in order to enforce the statistical model and reduce the number of possible misclassified cases.

According to the conducted carbonisation experiments, in order to obtain optimal comparison samples, hearths placed on the ground should be used, the grape seeds covered with at least 2 cm of topsoil and the fire kept burning for at least eight hours. In this way, it is possible to speed up the work and achieve a higher number of samples than in the muffle furnace.

Therefore, in order to improve the current knowledge of the domestication processes, it could be useful to apply the same approach to other plant species that are difficult to identify. In any event, with this work it was possible to demonstrate that a new powerful tool is now available to archaeobotanists who need to correctly identify charred grape seeds.
